# Race and Socioeconomic Status as Predictors of Willingness to Use Digital Mental Health Interventions or One-On-One Psychotherapy: National Survey Study

**DOI:** 10.2196/49780

**Published:** 2024-04-11

**Authors:** Lorenzo Lorenzo-Luaces, Akash Wasil, Corinne N Kacmarek, Robert DeRubeis

**Affiliations:** 1 Indiana University-Bloomington Bloomington, IN United States; 2 Center for AI Safety San Francisco, CA United States; 3 American University Washington, DC United States; 4 University of Pennsylvania Philadelphia, PA United States

**Keywords:** digital mental health, ethnicity, health disparities, internet-based CBT, cognitive behavioral therapy, intervention, mental health, mental health care, race, therapy

## Abstract

**Background:**

There is an ongoing debate about whether digital mental health interventions (DMHIs) can reduce racial and socioeconomic inequities in access to mental health care. A key factor in this debate involves the extent to which racial and ethnic minoritized individuals and socioeconomically disadvantaged individuals are willing to use, and pay for, DMHIs.

**Objective:**

This study examined racial and ethnic as well as socioeconomic differences in participants’ willingness to pay for DMHIs versus one-on-one therapy (1:1 therapy).

**Methods:**

We conducted a national survey of people in the United States (N=423; women: n=204; mean age 45.15, SD 16.19 years; non-Hispanic White: n=293) through Prolific. After reading descriptions of DMHIs and 1:1 therapy, participants rated their willingness to use each treatment (1) for free, (2) for a small fee, (3) as a maximum dollar amount, and (4) as a percentage of their total monthly income. At the end of the study, there was a decision task to potentially receive more information about DMHIs and 1:1 therapy.

**Results:**

Race and ethnicity was associated with willingness to pay more of one’s income, as a percent or in dollar amounts, and was also associated with information-seeking for DMHIs in the behavioral task. For most outcomes, race and ethnicity was not associated with willingness to try 1:1 therapy. Greater educational attainment was associated to willingness to try DMHIs for free, the decision to learn more about DMHIs, and willingness to pay for 1:1 therapy. Income was inconsistently associated to willingness to try DMHIs or 1:1 therapy.

**Conclusions:**

If they are available for free or at very low costs, DMHIs may reduce inequities by expanding access to mental health care for racial and ethnic minoritized individuals and economically disadvantaged groups.

## Introduction

### Overview

Systemic racism and classism are pervasive in the United States, including in mental health care, and current systems often fail to meet the needs of racial and ethnic minoritized individuals and those who are economically and socially disadvantaged [1]. For example, relative to non-Hispanic White individuals, individuals from racial and ethnic minoritized groups are less likely to be offered psychotherapy, more likely to be offered psychiatric medication as a first-line treatment, and more likely to be forcibly detained for mental health concerns [2,3]. The existence of these biases may, understandably, lead socially and economically disadvantaged individuals to distrust traditional mental health services [4,5], such as face-to-face, one-on-one therapy (1:1 therapy), in which an individual patient receives assessment and intervention from a specialty provider. Digital mental health interventions (DMHIs) leverage technology (eg, websites and apps) to provide mental health assessment or intervention. DMHIs such as internet-based cognitive behavioral therapy have demonstrated efficacy relative to control conditions such as waiting lists and care as usual and may have roughly similar efficacy to 1:1 therapy [6]. These interventions may have the potential to reduce the public health burden of psychopathology because both the general public and health providers appear to find them acceptable for use and dissemination [7-9].

Despite the promise of DMHIs, there is an ongoing debate about whether DMHIs can reduce racial and ethnic, as well as socioeconomic, inequities in access to mental health care. DMHIs may reduce economic barriers because most popular DMHIs offer a “freemium” model, in which users can access some content for free and pay to receive the full version of the intervention. Notably, even the “premium” versions of a DMHI are generally much less expensive than other forms of treatment. The 2 most popular smartphone apps for depression and anxiety, for example, require users to pay US $13-15 a month [10,11]. Furthermore, given that many popular DMHIs offer unguided self-help [10,12], DMHIs may appeal to individuals whose trust in traditional services has been undermined.

On the other hand, racial and ethnic minoritized individuals have been poorly represented in research on DMHIs [13]. Experts have also raised concerns that lower internet access, digital health literacy, awareness of digital health interventions, availability of culturally sensitive interventions, and ability to pay for digital health interventions may exacerbate existing inequities in access to care [14]. These factors may make DMHIs less appealing rather than more appealing to members of racial and ethnic minoritized groups and individuals from lower socioeconomic status [15-17]. Before touting DMHIs as having the potential to reduce disparities in access to mental health care, there should be evidence that these interventions are equally acceptable, or more acceptable, than traditional mental health services for racial and ethnic minoritized individuals or those from lower socioeconomic statuses. However, to our knowledge, no previous studies have examined interest in and willingness to pay for DMHIs among members of racial and ethnic minority groups or socioeconomically disadvantaged groups.

One way to study the acceptability of DMHIs and 1:1 therapy is to measure the self-reported willingness of individuals to engage with these interventions. While querying willingness to use as a measure of potential engagement is not equivalent to the more face valid option of offering DMHIs and 1:1 therapy to large numbers of individuals and capturing racial and ethnic and socioeconomic differences in engagement, it is more feasible. According to the Theory of Planned Behavior [18], attitudes, norms, and perceptions, such as self-reported willingness to use, can be used to predict many different types of behaviors, such as seeking mental health treatment [19,20]. Thus, the relationship between attitudes and willingness to use self-help interventions could predict future use. Understanding the demographics of individuals most likely to use DMHIs and 1:1 therapy can help target engagement efforts and, consequently, broaden the reach of these evidence-based self-help interventions to racially and ethnically marginalized groups.

### Objective

We conducted a nationally representative survey on participants’ willingness to use DMHIs and 1:1 therapy. Participants were adults living in the United States and recruited through the web-based survey research tool Prolific. Participants rated their willingness to use each treatment (1) for free, as well as their willingness to pay for the treatments (2) for a small fee, (3) as a maximum dollar amount, and (4) as a percentage of their total income. At the end of the study, we gave participants the option to engage in a behavior that we observed: information-seeking about DMHIs or 1:1 therapy. We also compare the relative willingness of participants to learn more about DMHI and 1:1 therapy.

## Methods

### Participants

Participants (N=423) were recruited through Prolific [21], a web-based survey platform. Participants were eligible if they were aged 18 years or older and lived in the United States. We obtained a sample of adults meant to be representative of the intersection of age, race and ethnicity, and sex-assigned at birth using US census data.

### Measures

#### Demographics

We collected information on age (in years), gender identity (male, female, and nonbinary), yearly income (in US dollars), highest educational attainment, race, and ethnicity. Race and ethnicity were combined and defined as Asian, Hispanic, non-Hispanic Black, non-Hispanic White, or other (eg, multiracial or Middle Eastern).

#### Internalizing Distress

We measured internalizing distress with the Kessler Psychological Distress Scale (K6) [22,23]. K6 is a 6-item scale that asks participants the frequency of distress symptoms (eg, depression and nervousness) they have experienced over the past month on a 4-point scale (0=none of the time and 4=all of the time). Scores can range from 0 to 24, with higher scores indicating higher distress. K6 has been demonstrated to have criterion validity [24] and was an internally consistent measure of internalizing distress in this sample (α=.87).

#### Willingness to Pay

First, participants received descriptions of unguided DMHIs and 1:1 therapy. DMHIs were described as “websites, computer programs, or smartphone apps” that “include information and exercises designed to help people learn skills that improve their mental health or well-being.” It was further described that in unguided DMHIs, “individuals learn content from a website or an app on their own. They do not have access to a coach or mentor.” 1:1 Therapy was defined as “counseling in which people receive support from a trained mental health professional who has completed a degree in counseling psychology, clinical psychology, or a related field.” It was further clarified that in “one-on-one therapy from a professional, one individual receives support from one therapist.”

Willingness to pay was evaluated with a series of different outcomes, which were presented in a randomized order by treatment (DMHI questions first vs 1:1 therapy questions first):

1. For free: Participants were asked to rate their agreement with the statement “I would be willing to use an unguided web-based self-help program or smartphone app for free.” For 1:1 therapy, a parallel question was used, replacing “an unguided web-based self-help program or smartphone app” with “weekly one-on-one therapy with a professional.” Responses were recorded on a 7-point Likert scale (1=strongly disagree and 7=strongly agree) with a higher score indicating a greater willingness to use.
2. Low cost: Participants were asked to rate their agreement with the statement “I would be willing to pay US $13 per month for an unguided web-based self-help program or smartphone app.” For 1:1 therapy, a parallel question was used, replacing “an unguided web-based self-help program or smartphone app” with “weekly one-on-one therapy with a professional” and replacing “US $13 per month” with “US $100 per month (US $25/session).” Responses were recorded on a 7-point Likert scale (1=strongly disagree and 7=strongly agree) with a higher score indicating a greater willingness to pay. The value for DMHIs was chosen to reflect the cost of premium versions of unguided DMHIs. For 1:1 therapy, the values were chosen to reflect the cost of therapy with insurance coverage.
3. Maximum dollar amount: Participants were asked to type “the maximum dollar amount” they would be “willing and able to pay” for “an unguided web-based self-help program or smartphone app” and for “one-on-one therapy with a professional”. Responses were recorded as dollar amounts starting from US $0 and with a maximum of US $10,000,000, although they were capped at US $800 for 1:1 therapy and US $60 for DMHIs.
4. Percentage income: Participants were asked to enter “the maximum percentage” of their monthly that they thought they would be willing and able to pay for “an unguided web-based self-help program or smartphone app” or “one-on-one therapy with a professional.” Responses were recorded as percentage amounts in a 0% to 100% range. We opted to ask participants this question, in addition to a maximum exact dollar amount, given evidence that response quality tends to be better with percentage metrics than when asking people to answer in dollar amounts [25].

### Statistical Analyses

The data and code for all analyses are available in the Open Science Framework website [26]. All analyses were performed in the R programming language [27] using R (version 4.3.1; R Core Team) with the R Studio GUI (version 2023.6.0.421; R Studio Team) [28]. A *P*<.05 was chosen as the criterion for statistical significance given the exploratory nature of the study. First, we present descriptive statistics to characterize the sample demographics by race and ethnicity. For categorical and ordered variables, we present the number of individuals endorsing each level of the variable. For continuous variables, we present means, SDs, and IQRs. Next, we report simple descriptive statistics (ie, median and IQR) on the various willingness to pay metrics by race and ethnicity.

To address potential differences in willingness to use DMHI and 1:1 therapy, we regressed the various willingness to use outcomes on race and ethnicity, educational attainment, and income, controlling for age and distress. For the ordinal outcomes (ie, agreement with willingness to use for free or for a small fee), the regressions were ordinal logistic regression [29]. For the percentage and raw dollar amount outcomes, we used linear regressions. For the binary outcome (ie, the decision to learn more or not about DMHI and 1:1 therapy), the regression of interest was a binary logistic regression predicting the selection of information (yes vs no). We verified that multicolinearity was low for the variables ultimately included in our model (ie, race and ethnicity, educational attainment, income, distress, and age). We also verified the results were not sensitive to influential cases and different modeling strategies (eg, using beta regression for the bounded percentage outcomes). Additionally, for the ordinal outcomes, we verified the proportional odds assumptions of ordinal regression were met for all the variables.

R packages were used for general programming needs (*conflicted* [30] and *tidyverse* [31]), to facilitate data cleaning and analysis (*MASS* [32], *effects* [33], *broom* [34], *psych* [35], *rstatix* [36], and *effectsize* [37]), and for making for tables (*gtsummary* [38], *labelled* [39], *kableExtra* [40], and *flextable* [41]) and figures (*ggpubr* [42], *gghalves* [43], and *scales* [44]). When examining willingness to pay as a maximum percentage of financial resources, we addressed outliers through winsorization. Responses that were >3 SDs above the mean were set to the value 3 SDs above the mean (6 values were winsorized for willingness to pay for unguided DMHIs and 9 values were winsorized for one-on-one psychotherapy).

### Ethical Considerations

The University of Pennsylvania Institutional Review Board approved this study (#843424). Participants provided written informed consent to take part in the study. The study consisted of a web-based survey lasting approximately 15 minutes. The study was described as being about attitudes toward different mental health interventions. Participants were informed that their data would be collected and analyzed in an anonymous fashion. Individuals were paid US $5 for their participation in the study.

## Results

### Demographics

**Figure 1:**
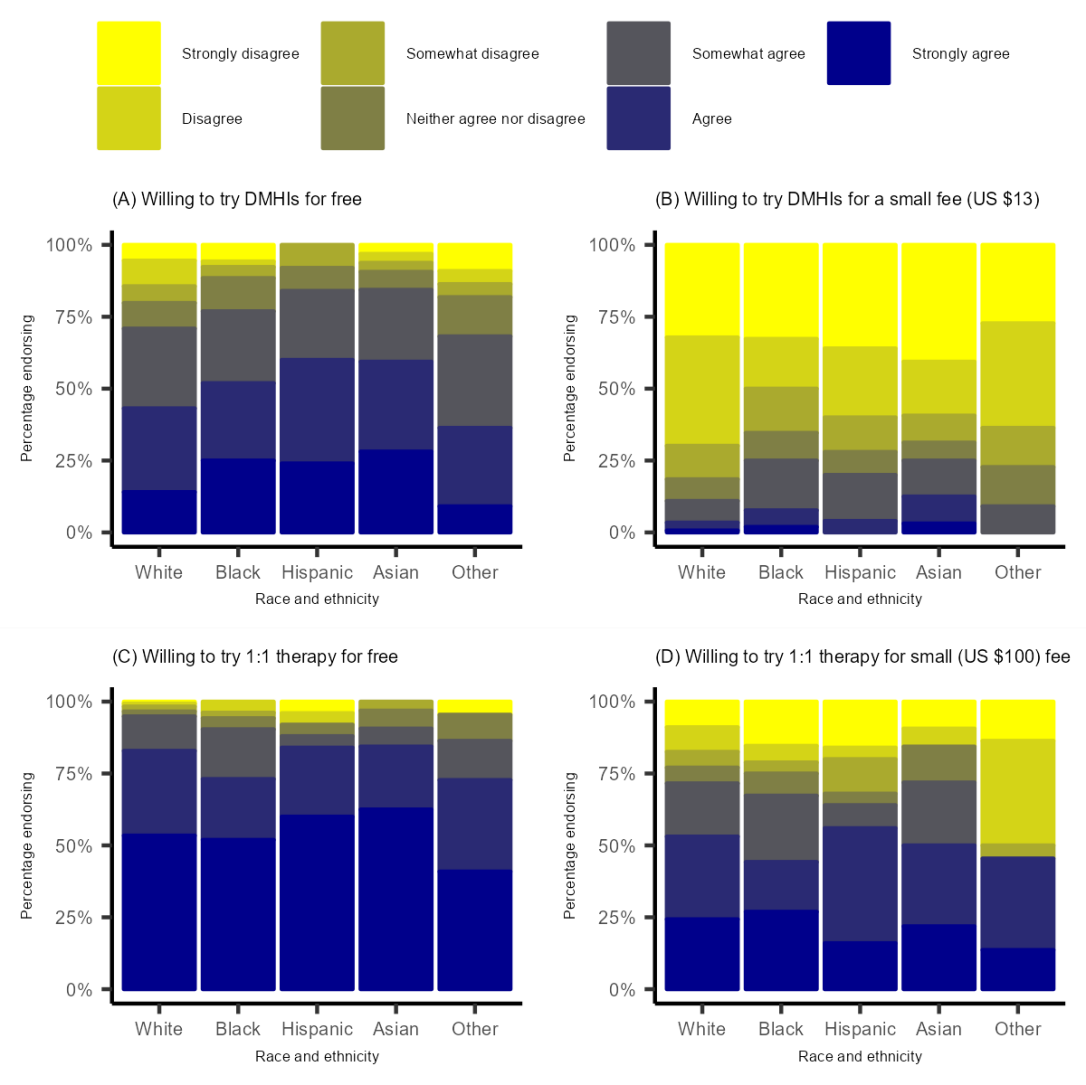
Willingness to use digital mental health interventions (DMHIs) or one-on-one therapy (1:1 therapy) for free or a low cost in a nationally representative sample of Prolific users in the United States (N=423). (A) Willing to try DMHIs for free; (B) willing to try DMHIs for a small fee (US $13); (C) willing to try 1:1 therapy for free; and (D) willing to try 1:1 therapy for a small fee (US $100).

**Figure 2:**
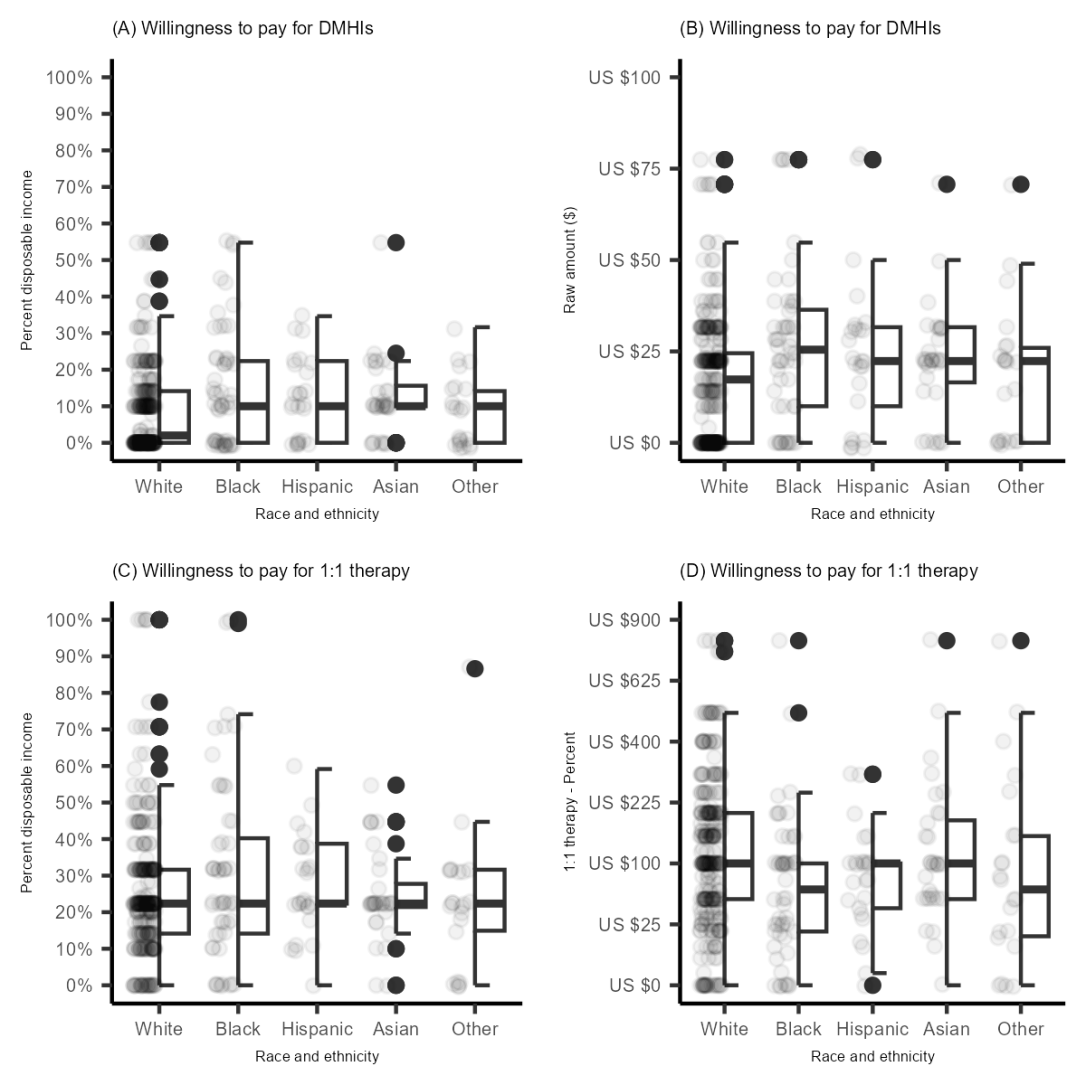
Willingness to use digital mental health interventions (DMHIs) or one-on-one therapy (1:1 therapy) as a percentage of income or a raw dollar amount in a nationally representative sample of Prolific users in the United States (N=423). (A) Willingness to pay for DHMIs as a percentage of income; (B) willingness to pay for DMHIs as a raw dollar amount; (C) willingness to pay for 1:1 therapy as a percentage of income; and (D) willingness to pay for 1:1 therapy as a raw dollar amount.

The sample appeared fairly representative of the United States in terms of age, gender identity, as well as race and ethnicity. There were differences between the racial and ethnic groups in age, income, and educational attainment (Table 1). Descriptive statistics for the various willingness to use and pay for DMHI and 1:1 therapy are presented in Figures 1 and 2, respectively. Willingness to use DMHIs was relatively high when they are described as being “free.” Participants were much less likely to say they would be willing to use DMHIs if they had to incur a small fee to do so. Across these outcomes, racial and ethnic differences suggested that relative to non-Hispanic White individuals, racial and ethnic minoritized individuals were more willing to use or pay for DMHIs. Similarly, willingness to use 1:1 therapy was very high when they are described as being “free.” Willingness dropped when participants were asked to pay a small monthly fee (ie, US $100 or $25 per session) for 1:1 therapy.

**Table 1:** Racial and ethnic differences in sociodemographic factors for adults in a nationally representative sample in the United States (N=423).

Characteristics					Non-Hispanic White (n=293)		Non-Hispanic Black (n=52)		Hispanic (n=25)		Asian (n=31)		Other (eg, multiracial and Middle Eastern) (n=22)		*P* value^a^	
Age (years), mean (SD)					47.15 (16.16)		43.19 (15.45)		33.56 (15.28)		37.45 (13.74)		47.18 (14.25)		<.001	
Income (in US $10,000), mean (SD)					7.45 (4.94)		5.94 (4.10)		5.80 (3.14)		7.35 (5.62)		6.07 (6.55)		.04	
**Gender, n (%)**																.99
	Man			49.83 (146)		46.15 (24)		52 (13)		51.61 (16)		50 (11)				
	Woman			47.78 (140)		51.92 (27)		44 (11)		48.39 (15)		50 (11)				
	Nonbinary			2.39 (7)		1.92 (1)		4 (1)		0 (0)		0 (0)				
**Educational attainment, n (%)**																<.001
		High school or less		10.24 (30)		15.38 (8)		4 (1)		6.45 (2)		4.55 (1)				
		Some college (eg, associate’s degree)		26.28 (77)		25 (13)		64 (16)		9.68 (3)		63.64 (14)				
		College graduate		38.23 (112)		42.31 (22)		24 (6)		67.74 (21)		18.18 (4)				
		Master’s degree or above		25.26 (74)		17.31 (9)		8 (2)		16.13 (5)		13.64 (3)				
**Sexual orientation, n (%)**																.56
			Heterosexual	86.01 (252)		86.54 (45)		76 (19)		80.65 (25)		90.91 (20)				
			LGBTQ+^b^	13.99 (41)		13.46 (7)		24 (6)		19.35 (6)		9.09 (2)				

^a^Kruskal-Wallis rank sum test; Fisher exact test for count data with simulated *P* value (based on 2000 replicates).

^b^LGBTQ+: lesbian, gay, bisexual, transgender, queer.

### Willingness to Try DMHIs or 1:1 Therapy, if Free or a Small Fee

Table 2 shows the results of ordinal logistic regressions predicting willingness to use DMHIs or 1:1 therapy, when described as for “free” or for a small fee (ie, US $13 for DMHI vs $25 a week for 1:1 therapy), from race and ethnicity, education, and income, controlling for sociodemographic factors and internalizing distress. Race and ethnicity did not predict willingness to try DMHIs or 1:1 therapy for free or for a small fee, although the differences suggested racial and ethnic minoritized individuals were more rather than less willing to try DMHIs. Educational attainment appeared associated with a greater willingness to try DMHIs or 1:1 therapy for free, or 1:1 therapy for a small fee, but the effects of educational attainment on willingness measured crossed the *P*<.05 threshold. Income was not associated with willingness to willingness to try DMHIs or 1:1 therapy for free. It was associated with a willingness to try DMHIs or 1:1 therapy for a small fee, although the effect was small (Table 2).

**Table 2:** Race and ethnicity and socioeconomic factors as predictors of willingness to try digital mental health interventions (DMHIs) versus one-on-one therapy (1:1 therapy) for free or a small fee in a nationally representative sample of US adults (N=423).

	Characteristics					DMHI					1:1 therapy		
							OR^a^ (95% CI)		*P* value		OR (95% CI)		*P* value
**Question 1: for free**													
		**Race and ethnicity**							.20				.70
				Asian		1.76 (0.88-3.52)				1.29 (0.60-2.92)			
				Hispanic		1.49 (0.69-3.25)				1.05 (0.44-2.59)			
				Non-Hispanic Black		1.58 (0.92-2.72)				0.81 (0.45-1.45_			
				Non-Hispanic White		—^b^				—			
				Other (eg, multiracial and Middle Eastern)		0.78 (0.36-1.71)				0.63 (0.28-1.45)			
		Distress (K6^c^)					1.00 (0.97-1.04)		.84		1.02 (0.99-1.06)		.20
		Age (years)					0.98 (0.97-0.99)		.002		0.99 (0.97-1.00)		.04
		**Educational attainment**							.08				.052
			High school or less			—				—			
			Some college (eg, associate’s degree)			2.10 (1.09-4.04)				1.21 (0.59-2.43)			
			College graduate			2.26 (1.21-4.23)				1.35 (0.68-2.65)			
			Master’s degree or above			1.92 (0.96-3.84)				2.36 (1.10-5.06)			
		Income (in US $10,000)					0.97 (0.93-1.01)		.10		0.99 (0.95-1.03)		.50
**Question 2: for a small fee**													
		**Race and ethnicity**							.30				.60
				Asian		1.30 (0.61-2.73)				0.87 (0.44-1.72)			
				Hispanic		1.44 (0.63-3.25)				0.89 (0.41-1.95)			
				Non-Hispanic Black		1.75 (1.00-3.06)				1.02 (0.59-1.76)			
				Non-Hispanic White		—				—			
				Other (eg, multiracial and Middle Eastern)		1.37 (0.63-2.93)				0.54 (0.25-1.18)			
		Distress (K6^c^)					0.97 (0.93-1.00)		.051		1.02 (0.98-1.05)		.30
		Age (years)					1.01 (0.99-1.02)		.40		0.99 (0.98-1.00)		.20
		**Educational attainment**							.15				.06
					High school or less	—				—			
					Some college (eg, associate’s degree)	0.74 (0.38-1.45)				1.51 (0.79-2.88)			
					College graduate	0.90 (0.48-1.72)				1.48 (0.79-2.79)			
					Master’s degree or above	0.54 (0.26-1.09)				2.43 (1.21-4.89)			
		Income (in US $10,000)					1.05 (1.01-1.09)		.02		1.10 (1.06-1.14)		<.001

^a^OR: odds ratio.

^b^Not available.

^c^K6: Kessler Psychological Distress Scale.

### Willingness to Try DMHIs or 1:1 Therapy, as Percent of Income or Raw Dollar Amount

Tables 3 and 4 show the results of regressions predicting willingness to pay for DMHIs or 1:1 therapy as a percentage of monthly disposable income (Table 3) or as a raw dollar amount (Table 4). Compared to non-Hispanic White adults, racial and ethnic minoritized individuals were willing to pay more for DMHIs as a percentage of their income (Table 3) or as a raw dollar amount (Table 4). Examining the pairwise contrasts revealed that these differences were the largest and statistically significant when comparing non-Hispanic Black adults to non-Hispanic White adults for a percentage of their income (*P*<.001; Table 3) or a raw dollar amount (*P*<.001; Table 4), as well as for Hispanic adults for a raw dollar amount (*P*=.04). The other minoritized groups, Asian and Other (eg, multiracial and Middle Eastern), appeared somewhat more willing to pay but the differences were small and not statistically significant. Educational attainment was inconsistently associated with willingness to pay. For example, it appeared unrelated to willingness to pay for either DMHIs or 1:1 therapy (Table 3). However, when willingness was assessed on a dollar metric, greater educational attainment was associated with a greater willingness to pay for 1:1 therapy, with effects most pronounced for college graduates and those with a master’s degree or greater educational attainment. By contrast, although educational attainment was associated with greater willingness to pay for DMHIs, the pairwise contrasts comparing the educational groups to those with a high school degree or less were small and not statistically significant. Income was associated with a greater willingness to pay for both DMHIs and 1:1 therapy in raw dollar amounts (Table 4) but not as a percentage of income.

**Table 3:** Race and ethnicity and socioeconomic factors as predictors of the percent of income willing to pay for face-to-face therapy in a nationally representative sample of US adults (N=423).

Characteristics			Percent income willing to pay for DMHI^a^										Percent income willing to pay for 1:1 therapy							
			B (unstandardized; SE)		*t* test (*df*=412)		β (standardized; 95% CI)		*P* value			B (unstandardized; SE)		*t* test (*df*=412)		β (standardized; 95% CI)		*P* value		
Intercept			1.53 (0.35)		4.41		0.00 (–0.09 to 0.09)		<.001			2.89 (0.53)		5.43		0.00 (–0.09 to 0.09)		<.001		
**Race and ethnicity**										.003									.30	
	Asian	0.32 (0.23)		1.40		0.07 (–0.03 to 0.17)		.16			–0.11 (0.35)			–0.32	–0.02 (–0.11 to 0.08)		.75			
	Hispanic	0.32 (0.26)		1.25		0.06 (–0.04 to 0.16)		.21			0.06 (0.40)			0.16	0.01 (–0.09 to 0.11)		.88			
	Non-Hispanic Black	0.69 (0.18)		3.82		0.19 (0.09 to 0.28)		<.001			0.56 (0.28)			2.02	0.10 (0.00 to 0.20)		.04			
	Non-Hispanic White	—^b^		—		—		—			—			—	—		—			
	Other (eg, multiracial and Middle Eastern)	0.01 (0.27)		0.05		0.00 (–0.09 to 0.10)		.96			–0.21 (0.41)			–0.51	–0.03 (–0.12 to 0.07)		.61			
Distress (K6^c^)			0.01 (0.01)		0.66		0.04 (–0.07 to 0.14)		.51			0.03 (0.02)		1.83		0.10 (–0.01 to 0.21)		.07		
Age (years)			–0.01 (0.00)		–1.32		–0.07 (–0.18 to 0.04)		.19			–0.01 (0.01)		–1.51		–0.09 (–0.20 to 0.03)		.13		
**Educational attainment**										.28									.88	
	High school or less	—		—		—		—			—			—	—		—			
	Some college (eg, associate’s degree)	–0.12 (0.22)		–0.54		–0.05 (–0.21 to 0.12)		.59			–0.05 (0.34)			–0.14	–0.01 (–0.18 to 0.15)		.89			
	College graduate	–0.16 (0.21)		–0.77		–0.07 (–0.24 to 0.10)		.44			–0.19 (0.32)			–0.59	–0.05 (–0.22 to 0.12)		.56			
	Master’s degree or above	–0.24 (0.23)		–1.05		–0.08 (–0.24 to 0.07)		.29			0.05 (0.35)			0.13	0.01 (–0.15 to 0.17)		.90			
Income (in US $10,000)			0.01 (0.01)		1.09		0.06 (–0.04 to 0.15)		.28			–0.00 (0.02)		–0.15		–0.01 (–0.11 to 0.09)		.88		

^a^DMHI: digital mental health intervention.

^b^Not available.

^c^K6: Kessler Psychological Distress Scale.

**Table 4:** Race and ethnicity and socioeconomic factors as predictors of the raw dollar amount willing to pay for face-to-face therapy in a nationally representative sample of US adults (N=423).

Characteristics				Dollar amount willing to pay for DMHI^a^											Dollar amount willing to pay for 1:1 therapy							
				B (unstandardized; SE)		*t* test (*df*=412)		β (standardized; 95% CI)		*P* value			B (unstandardized; SE)				*t* test (*df*=412)		β (standardized; 95% CI)		*P* value	
Intercept				3.26 (0.51)		6.37		0.00 (–0.09 to 0.09)		<.001			8.45 (1.67)				5.06		–0.00 (–0.09 to 0.09)		<.001	
**Race**											.002											.14
	Asian		0.45 (0.34)		1.32		0.07 (–0.03 to 0.16)		.19			–0.72 (1.10)				–0.65		–0.03 (–0.12 to 0.06)		.52		
	Hispanic		0.78 (0.38)		2.04		0.10 (0.00 to 0.20)		.04			–1.36 (1.25)				–1.09		–0.05 (–0.15 to 0.04)		.28		
	Non-Hispanic Black		0.97 (0.26)		3.68		0.18 (0.08 to 0.28)		<.001			–2.17 (0.87)				–2.51		–0.12 (–0.21 to –0.03)		.01		
	Non-Hispanic White		—^b^		—		—		—			—				—		—		—		
	Other (eg, multiracial and Middle Eastern)		0.30 (0.39)		0.76		0.04 (–0.06 to 0.13)		.45			–0.57 (1.28)				–0.44		–0.02 (–0.11 to 0.07)		.66		
Distress (K6^c^)				–0.02 (0.02)		–0.94		–0.05 (–0.16 to 0.06)		.35			0.05 (0.05)				0.85		0.04 (–0.06 to 0.15)		.40	
Age (years)				–0.01 (0.01)		–1.49		–0.08 (–0.19 to 0.03)		.14			–0.03 (0.02)				–1.52		–0.08 (–0.19 to 0.02)		.13	
**Educational attainment**											.02											<.001
	High school or less		—		—		—		—			—				—		—		—		
	Some college (eg, associate’s degree)		0.20 (0.32)		0.63		0.05 (–0.11 to 0.21)		.53			1.19 (1.06)				1.12		0.09 (–0.07 to 0.25)		.26		
	College graduate		0.39 (0.31)		1.26		0.11 (–0.06 to 0.28		.21			2.77 (1.02)				2.71		0.22 (0.06 to 0.39)		.01		
	Master’s degree or above		0.16 (0.34)		0.46		0.04 (–0.12 to 0.19)		.65			3.12 (1.12)				2.80		0.21 (0.06 to 0.36)		.01		
Income (in US $10,000)				0.04 (0.02)		2.35		0.12 (0.02 to 0.22)		.02			0.31 (0.06)				5.26		0.25 (0.16 to 0.35)		<.001	

^a^DMHI: digital mental health intervention.

^b^Not available.

^c^K6: Kessler Psychological Distress Scale.

### Decision to Learn More About DMHIs and 1:1 Therapy

Race and ethnicity predicted the decision to learn more about DMHIs (*P=*.02) and 1:1 therapy (*P=*.02; Table 5). Compared to non-Hispanic White individuals, non-Hispanic Black individuals, Hispanic individuals, and individuals classified as “Other” (eg, multiracial and Middle Eastern) were more likely to choose to learn more about both DMHIs and 1:1 therapy. Asian individuals were somewhat less likely to choose to learn about DMHIs but somewhat more likely to learn about 1:1 therapy. Educational attainment was associated with a decision to learn more about DMHIs (*P=*.02) but not 1:1 therapy (*P=*.30). Income was not associated with the decision to learn about DMHIs (*P*=.92) or 1:1 therapy (*P*=.20)

**Table 5:** Decision to learn more about digital mental health interventions (DMHIs) or one-on-one therapy (1:1 therapy) in a nationally representative sample of respondents (N=423).

Characteristics			DMHIs				1:1 therapy		
			OR^a^ (95% CI)		*P* value		OR (95% CI)		*P* value
**Race and ethnicity**					.02				.02
	Asian	0.85 (0.29-2.18)				1.27 (0.38-3.60)			
	Hispanic	5.00 (1.85-13.4)				1.87 (0.47-6.23)			
	Non-Hispanic Black	1.85 (0.88-3.78)				4.03 (1.81-8.85)			
	Non-Hispanic White	—^b^				—			
	Other (eg, multiracial and Middle Eastern)	1.26 (0.33-3.94)				1.79 (0.38-6.33)			
Distress (K6^b^)			1.12 (1.07-1.17)		<.001		1.13 (1.07-1.19)		<.001
Age (years)			1.01 (0.99-1.03)		.40		0.98 (0.96-1.01)		.14
**Educational attainment**					.02				.30
	High school or less	—				—			
	Some college (eg, associate’s degree)	0.96 (0.35-2.83)				1.08 (0.35-3.58)			
	College graduate	2.45 (0.97-6.90)				1.63 (0.58-5.18)			
	Master’s degree or above	2.13 (0.77-6.42)				2.28 (0.73-7.84)			
Income (in US $10,000)			1.00 (0.95-1.06)		.92		1.04 (0.98-1.11)		.20

^a^OR: odds ratio.

^b^K6: Kessler Psychological Distress Scale.

## Discussion

### Principal Findings

Across several metrics, we found that members of racial and ethnic minoritized groups were either willing to pay more for DMHIs than non-Hispanic White participants or had similar levels of willingness. These differences were especially large when comparing non-Hispanic Black and Hispanic adults to non-Hispanic White adults. Race and ethnicity was not a consistent predictor of willingness to use 1:1 therapy. The other sociodemographic factors had somewhat more predictable relation to willingness to use DMHIs and 1:1 therapy. More educated individuals were more likely to say they would pay for 1:1 therapy or try DMHIs if the interventions were free. Income was associated with a higher willingness to pay for services, although the associations were less consistent.

### Limitations

While we recruited a nationally representative sample of individuals, our sample size (N=423) may have been too low to detect small associations between sociodemographic factors and willingness to use or pay that would nonetheless be of interest. Additionally, we did not measure participants’ experiences with DMHI, which was likely low, or 1:1 therapy, which was likely higher overall [45]. Finally, while our results are informative in terms of capturing attitudes toward DMHIs and 1:1 therapy, the data we collected are self-reports of a prospective, relatively low burden behavior and may not reflect the decision to use mental health services in the real world. A notable bias of self-report data includes a limitation on self-knowledge (eg, individuals may not be aware of how they would act in the event they needed to choose between DMHIs and 1:1 therapy) as well as a sometimes low correspondence between self-report and behavior [46]. Other possible biases include overly positive responding and social desirability (eg, individuals presenting themselves as more willing to seek treatment than they are likely to do in real life). It is worth noting that for these biases to affect our results, which pertain to racial or ethnic differences, the biases would have to operate differentially across the groups we considered.

Several strengths of this study are worth noting. First, we operationalized willingness to use and willingness to pay in a variety of ways that support a similar conclusion: that racial and ethnic minoritized individuals are willing to use and pay for DMHIs. Additionally, we measured a behavioral proxy of treatment seeking—a willingness to learn more about different interventions—to further contextualize our results. Finally, we explored racial and ethnic and socioeconomic differences in a diverse sample of individuals.

### Comparison With Previous Work

Our results suggest that racial and ethnic minoritized individuals are roughly equally likely, or perhaps even more likely, to use DMHIs than non-Hispanic White individuals. Previous work has suggested that racial discrimination in health care may lead members of minority groups to lose trust in health care systems [4,5]. Given this history of discrimination and inequitable treatment in health care settings, alternatives to traditional services—such as unguided digital self-help interventions—may be especially appealing to members of socioeconomically disadvantaged groups and racial and ethnic minoritized individuals. An implication of these findings is that when low levels of engagement are seen in DMHIs, it is unlikely that these effects are due to racial and ethnic minoritized groups having an overall low willingness to use DMHIs and may instead reflect access issues (eg, DMHI advertisement not reaching racial and ethnic minoritized individuals). Additionally, our results revealed a notable reaction to costs: around two-thirds (65.2%, 276/423) of individuals are unwilling to use DMHIs if there is even a small cost associated with the interventions. These findings reiterate how costs may be a barrier to mental health treatment and support other calls to enhance the accessibility of DMHIs for racial and ethnic minoritized individuals [14,47]. Recent research has compared strategies to increase the adoption of DMHIs in health care [48]. Future research can explore the effectiveness of these strategies on increasing adoption of DMHIS, specifically, among racial and ethnic minoritized individuals.

### Conclusions

These findings do not support the concern that DMHIs appeal selectively to racial and ethnic majority members and wealthier individuals. Instead, racial and ethnic minoritized individuals indicated a greater willingness to pay for DMHIs, and income was inconsistent with willingness to pay for DMHIs. The promotion of effective and affordable DMHIs could be an important way to reduce inequities and expand access to mental health care for socially and economically disadvantaged groups. Importantly, our results suggested that the willingness to use interventions when they are delivered for free is quite high for both DMHIs and 1:1 therapy.

## Abbreviations

1:1 therapy: one-on-one therapy
DMHI: digital mental health intervention
K6: Kessler Psychological Distress Scale

## Data Availability

The data sets generated during and/or analyzed during this study are available in the Open Science Foundation website [26].
